# The effect of a single dose of prednisolone in dogs envenomated by *Vipera berus* – a randomized, double-blind, placebo-controlled clinical trial

**DOI:** 10.1186/s12917-015-0352-6

**Published:** 2015-02-26

**Authors:** Erika Brandeker, Anna Hillström, Sofia Hanås, Ragnvi Hagman, Bodil Ström Holst

**Affiliations:** Evidensia Södra Djursjukhuset, Månskärsvägen 13, SE-141 75 Kungens Kurva, Sweden; Department of Clinical Sciences, Swedish University of Agricultural Sciences, Box 7054, , SE-750 07 Uppsala, Sweden

**Keywords:** *Vipera berus*, Snake bite, Dog, Prednisolone, C-reactive protein, Inflammation, Clinical trial

## Abstract

**Background:**

Treatment with glucocorticoids after snakebite in dogs is controversial and randomized clinical studies are missing. The objective of this study was to investigate the effect of a single dose of prednisolone in dogs envenomated by *Vipera berus* in a double-blind placebo-controlled study, after exclusion of dogs treated with antivenom. The two treatment groups were compared regarding clinical status and clinicopathological test results. A total of 75 dogs bitten by *Vipera berus* within the previous 24 hours were included. Clinical assessment, blood sampling and measurement of the bitten body part were done at admission (Day 1), after 24 hours (Day 2) and at a re-examination (Re-exam) after 10–28 days. Dogs were given prednisolone 1 mg/kg bodyweight (PRED) or saline (PLACEBO) subcutaneously in a randomized, double-blind clinical trial. Dogs were examined clinically and mental status and extent of edema were described. Furthermore, appetite, vomiting, diarrhea, cardiac arrhythmia and death were recorded. Concentrations of C-reactive protein (CRP) and high sensitivity cardiac Troponin I (cTnI), hematology variables and Prothrombin time (PT) were determined. Systemic inflammation was defined as present if CRP > 35 mg/l.

**Results:**

None of the dogs died during the study period. The mental status was reduced in 60/75 (80%) of dogs on Day 1, compared to 19/75 (25%) on Day 2. The proportion of dogs with no or only mild edema increased significantly from Day 1 to Day 2. About one-third of the dogs developed gastrointestinal signs during the study period. Cardiac arrhythmia was uncommon. Clinicopathological changes included increased total leucocyte count, CRP and troponin concentration on Day 2. The cTnI concentration was increased in dogs with systemic inflammation, compared to dogs without systemic inflammation. A single dose of prednisolone did not significantly affect any of the clinical or clinicopathological parameters studied, except for a higher monocyte count on Day 2 in dogs that had received prednisolone treatment.

**Conclusion:**

The results of the present study do not support routine administration of a single dose of prednisolone 1 mg/kg subcutaneously in dogs bitten by *Vipera berus*.

## Background

*Vipera berus* (*V. berus*) is the only venomous snake species in Sweden and every year about 1500–2000 dogs require veterinary care because of snake envenomation (Olsson P-E, Agria Pet Insurance Co, personal communication, 2012). The viper venom has proteolytic, fibrinolytic, anticoagulant and phospholipase A_2_ effects [1,2]. Clinical findings in dogs bitten by *V. berus* include local edema, pain, reduced mental status and cardiac arrhythmia [3-6]. The reported mortality ranges from 0 to 4.6% [3,4,6]. Common hematological findings in dogs bitten by *V. berus* are leucocytosis and increased hematocrit [2,3]. Minor liver injury has also been found [4] as well as myocardial cell damage [7,8]. Biochemical evidence of systemic inflammation has been described and was associated with biochemical signs of myocardial injury [7], but the degree of systemic inflammation has so far not been related to clinical status.

Therapy for dogs bitten by *V. berus* is mainly supportive, and includes intravenous (IV) fluids, opioid analgesics, glucocorticoids and antimicrobial drugs [4,6]. Specific treatment with antivenom can be recommended for dogs with moderate to severe clinical signs and may improve the general condition [5]. Treatment with glucocorticoids after snakebite in both humans and dogs is common but still controversial [4,6,9-12]. In humans bitten by *V. berus*, glucocorticoid treatment is only recommended in cases of acute allergic reactions against the venom, bronchospasm, or serum sickness after antivenom administration [10].

The aim of the present study was to describe the effect of a single dose of prednisolone 1 mg/kg subcutaneously in dogs bitten by *V.berus* in a prospective, double-blind placebo-controlled clinical trial. The two treatment groups were compared regarding clinical status and clinicopathological test results with special focus on inflammation.

## Methods

### Dogs

The study was done between April 2011 and September 2012 at two Swedish referral animal hospitals in central Sweden: Evidensia Södra Djursjukhuset (Animal Hospital 1) and Evidensia Specialistdjursjukhuset Strömsholm (Animal Hospital 2). Inclusion criteria were a reported snake bite (observation of a snake, fang marks or a very high suspicion of a snake bite) within the previous 24 h and presence of clinical signs typical of envenomation by *V. berus* (sudden development of local swelling and pain in the area of the suspected bite) within 2 h after the bite. Exclusion criteria were treatment with glucocorticoids or nonsteroidal antiinflammatory drugs (NSAID), pregnancy, vaccination within the last 2 weeks or renal disease, diabetes mellitus or hyperadrenocorticism. Dogs treated with antivenom were excluded. Other ongoing inflammatory disease was not an exclusion criterion but all medical records were reviewed retrospectively with respect to reported health status. One dog suffered from acute mastitis and was excluded from the CRP analysis.

At Animal Hospital 1, all owners arriving with a dog that met the inclusion criteria were asked if they wanted to participate in the study and all dogs were recorded, even those not enrolled in the study. In total 141 dogs met the inclusion criteria but 90 dogs were excluded because of treatment with glucocorticoids or NSAID (n = 17), pregnancy (n = 2), vaccination (n = 1), declination of the owner (n = 11), no further hospital stay after the initial examination (n = 5), negligence from the attending veterinarian to include the dog (n = 49) or treatment with antivenom (n = 5). Thus, in total 51 dogs were included at Animal Hospital 1 and 24 dogs at Animal Hospital 2. A total of 75 dogs were thus included in the study.

### Clinical assessments

All dogs were clinically examined by the attending veterinary surgeon on presentation (Day 1), after 24 h ± 5 h at Animal Hospital 1 or after 24 h ± 12 h at Animal Hospital 2 (Day 2), and at a follow-up visit after 10 to 28 days (Re-exam). At Animal Hospital 1 the results from the clinical examination were recorded both in a study protocol and in the medical records, whereas the information at Animal Hospital 2 was retrieved mostly from medical records due to incomplete data in the study protocol. Presence of diarrhea was determined retrospectively from the medical records. The time elapsed from the snake bite until the dog was clinically examined was recorded. Measurements of the bitten body parts were performed to assess the edema. If the dog was bitten on the nose, lips or in the face, the circumference of the middle of the muzzle was measured. If the dog was bitten in a paw or leg, a measurement was done where the dog was most swollen and this spot was marked with color or by shaving the fur over the area. A pilot study was performed at Animal Hospital 1 to test the measurement procedure. Fifteen veterinarians measured the muzzle and front paw of 2 healthy dogs (1 Labrador Retriever and 1 Jack Russel Terrier). The estimated accuracy of measurements was ± 0.5 cm (unpublished data). The circumference on Day 1 and Day 2 were compared to the circumference at Re-exam. At Re-exam no edema persisted and the circumference at Re-exam was used as a baseline to calculate the increase in percentage on the two first examination occasions. The edema was described as absent, mild, moderate or severe. Medical records from Day 1 until the day of Re-exam were studied, and presence of appetite, vomiting, diarrhea cardiac arrhythmia or death was recorded.

In 54 dogs Re-exam was done, but 21 dogs were lost to this follow-up examination. The owners of 6 of the 21 dogs were interviewed by telephone and reported that their dogs were recovering.

### Design of the clinical trial

Each dog was given a subcutaneous (SC) injection of either prednisolone^a^ (PRED) 1 mg/kg bodyweight (bw) or 0.9% NaCl (PLACEBO) by a technician in a dose of 0.1 ml/kg bw, according to a premade randomization list. The injection was given at admission, immediately after clinical examination and blood sampling were done. Neither the veterinary surgeon in charge nor the dog owner was aware of whether the dog received PRED (n = 38) or PLACEBO (n = 37) treatment. Dogs were further treated as decided by the veterinary surgeon in charge of the patient. The management of the cases was not affected by participation in the study, except that administration of glucocorticoids was not permitted. All dogs were treated with IV fluid and opioid analgesics (methadone or buprenorphine) according to individual need as judged by the veterinary surgeon in charge.

The main outcome variables for the clinical trial were mental status and edema. Further, differences between the PRED and the PLACEBO group regarding appetite, vomiting, diarrhea, cardiac arrhythmia and death were investigated. In addition, concentrations of CRP, high sensitivity cardiac Troponin I (cTnI), hematology variables and Prothrombin time (PT) were compared between the groups.

### Blood sampling and laboratory analyses

Blood was obtained from the cephalic or saphenous vein and collected in nonadditive (serum) and EDTA tubes on Day 1, 2 and at Re-exam. Serum samples were centrifuged 30 min after collection (5000 g, 5 min) and stored in cryotubes^b^ at −20°C for a maximum of 5 months. Samples were then transferred to −80°C where they were stored until analysis after a maximum of 24 (CRP) and 36 (cTnI) months, respectively. The complete blood count (CBC) was determined with fresh blood using an automated impedance hematology analyzer^c^ [13] at Animal Hospital 1, and an automated laser-based hematology analyzer^d^ [14,15] at Animal Hospital 2. After analysis, samples were centrifuged and plasma stored as for serum until PT analysis after a maximum of 24 months. Blood smears from Day 1 and Day 2 from dogs admitted to Animal Hospital 1 were evaluated, and a manual differential count was performed. Differential and platelet counts were reported only in dogs that had a manual differential count performed (i.e. admitted to Animal Hospital 1). C-reactive protein was measured with a previously validated canine-specific immunoturbidimetric assay^e^ on a fully automated chemistry/immunoassay analyzer^f^ [16]. The limit of quantification for this assay was 6.8 mg/l and lower results were reported as <6.8 mg/l. Systemic inflammation was defined as CRP > 35 mg/l [17].

Cardiac Troponin I (cTnI) was analyzed with a commercially available high-sensitivity immunoassay^g^ validated for use in dogs [18]. A combined prothrombin time (PT) reagent was used for measuring activity of coagulation factors II, VII and X^h^ . The reference range of the PT test was < 25 s and results below 25 s were reported as < 25 s.

### Statistical analysis

Statistical analyses and calculations were performed using the commercial software R 3.0.1 and in Minitab (Minitab Inc. State College, PA, USA). Descriptive statistics were done for all continuous variables and included mean, median, and standard deviation (SD). Spearman’s rank correlation coefficient (r_s_) was used to assess bivariate relationship amongst variables. McNemar’s test was used to compare the mental status and swelling on Day 1 with the same findings on Day 2. The chi-square proportion test was used to evaluate the differences of the distribution of clinical parameters (mental status, appetite, arrhythmia, vomiting, and diarrhea) between the PRED and PLACEBO groups. The unequal variance *t*-test was conducted to evaluate differences in WBC, swelling, duration of hospitalization, body weight and time elapsed from snake bite between the PRED and PLACEBO group. The Mann–Whitney *U*-test was used to evaluate differences in variables that were not normally distributed, which included CRP, hematocrit, troponin and PT. The level of statistical significance was set to p < 0.05.

### Ethical approval

The study was approved by the Local Ethical Committee on Animal Experiments (C31/11), and the Swedish Board of Agriculture (31-13905/10) and was approved as a clinical trial by the Swedish Medical Products Agency (152:2011/3282). Written owner consent was obtained for all dogs prior to inclusion in the study.

## Results

### Dogs

The 75 studied dogs represented 42 different breeds. There were 34 males, 35 females, 3 neutered males and 3 spayed females. The median age was 3 years (IQR 1–6) and the median weight 20.5 kg (IQR 11.8-28.0). The median time from snake bite to arrival at the animal hospital was 2.5 h (IQR 1.9-3.6), with a median duration of hospitalisation of 2 days (IQR 1.9-3.6). In total 62 dogs were bitten in the head region and 13 in a limb region.

### Clinical status

On Day 1 the mental status was normal in 15 dogs, mildly reduced in 37 dogs and moderately reduced in 22 dogs. The edema was considered to be mild in 10 dogs, moderate in 47 dogs and severe in 10 dogs. The appetite was not possible to describe accurately at arrival on Day 1. Cardiac arrhythmia was detected in 1 dog.

On Day 2, significantly more dogs (n = 56, p < 0.0001) had a normal mental status compared to Day 1. The mental status was mildly reduced in 17 dogs. Edema was determined as absent in 3 dogs, mild in 25 dogs, moderate in 29 dogs and severe in 11 dogs. Significantly more dogs had no or only mild edema Day 2 compared to Day 1 (p = 0.0013). At least 20 dogs (Data not available from all dogs included at Animal Hospital 2) had a reduced appetite. Cardiac arrhythmia was detected in 1 dog (not the same dog as that in which cardiac arrhythmia was detected on Day 1).

Vomiting was reported in 17 dogs and diarrhea in 14 dogs during the study period. In total, gastrointestinal signs (vomiting and diarrhea) were reported in 23 of 75 dogs. The edema had disappeared and the mental status was normal in all dogs by the time of Re-exam. None of the 75 dogs died during the study period.

### Clinicopathological parameters

#### C-reactive protein (CRP)

The median CRP concentration was < 6.8 mg/l on Day 1. Of 49 dogs that were presented at the animal hospital within 4 h after the snake bite, all except 3 had CRP concentration < 6.8 mg/l at the arrival, whereas the 11 dogs that arrived ≥ 4 h after the snake bite had a median (IQR) CRP concentration of 24 (17–28) mg/l. CRP concentration on Day 1 was positively correlated with time elapsed from bite to admission (r_s_ = 0.39, p < 0.01). CRP concentration increased to a median (IQR) concentration of 74 mg/l (41–101) on Day 2 (p < 0.001). At this time (Day 2), the CRP concentration was positively correlated to degree of swelling for the 33 dogs for which data on both variables were available (r_s_ = 0.38, p = 0.04), but CRP concentration did not differ between dogs with normal and dogs with decreased mental status (p = 0.45). Systemic inflammation was present in 39/49 (80%) of the dogs on Day 2. At Re-exam, the CRP concentration was < 6.8 mg/l in 42/46 (91%) dogs, and no dog had CRP concentration > 35 mg/l (systemic inflammation).

#### Prothrombin time (PT) and hematology

PT was within the reference range (<25 s) on Day 1 (46 samples) and Day 2 (38 samples) for all dogs except 3 dogs on Day 1 (25.6, 30.0 and 25.5 s) and 3 dogs on Day 2 (29.4, 44.5, 25.2 s). Of these dogs, 2 had increased PT on both Day 1 and Day 2. On Re-exam (35 samples) PT was within reference range for all tested dogs. The median (IQR) hematocrit was 47 (42–52)% on Day 1 (69 samples), 40 (34–44.5)% on Day 2 (49 samples) and 43 (39.5-48)% on Re-exam (41 samples). The hematocrit was significantly lower on Day 2 (p < 0.01) and Re-exam (p = 0.01) than on Day 1. The median (IQR) WBC was 12.3 (10.5-16.4) × 10^9^/l on Day 1 (71 samples). On Day 2, the median (IQR) WBC was significantly increased to 14.9 (12.4-18.9) × 10^9^/l, p = 0.01) (49 samples) compared to Day 1. At Re-exam (50 samples), the median (IQR) WBC numbers had decreased compared to both Day 1 (p < 0.01) and Day 2 (p < 0.01), to 9.6 (8.1-11.4) × 10^9^/l. An inflammatory leucogram, defined as band neutrophils > 1.0 × 10^9^/l, was present in 10/38 (26%) blood smears on Day 1, and in 7/32 (22%) blood smears on Day 2. Platelet numbers were within the reference range for healthy dogs in all investigated samples.

#### Cardiac troponin I (cTnI)

The median (IQR) cTnI concentration was 0.019 (0.010-0.058) μg/l on Day 1 (55 samples). On Day 2, the concentration of cTnI had increased significantly to 0.043 (0.021-0.29) μg/l (p = 0.001) (49 samples) and was at Re-exam 0.013 (0.007-0.023) μg/l (46 samples), which was significantly lower than both on Day 1 (p = 0.008) and on Day 2 (p < 0.001).

Dogs with systemic inflammation on Day 1 and Day 2 had a significantly (p < 0.01) higher median cTnI concentration (0.066 ng/ml) than dogs without systemic inflammation (0.021 ng/ml). CRP concentrations were significantly correlated with troponin concentrations at Day 2 (r_s_ = 0.35, p = 0.02), but not on Day 1 (r_s_ = −0.12, p = 0.38) or at Re-exam (r_s_ = 0.03, p = 0.8).

### Clinical trial

#### Dogs

The two groups did not differ significantly (p > 0.05) in the distributions of breed, age, body weight, sex or localization of the bite (Table 1). Fifty-four of the dogs arrived to the hospital less than 4 h after the snake bite, whereas 15 dogs arrived between 4 and 24 h after the bite had occurred. For 5 dogs detailed information about time elapsed from the bite was missing.

**Table 1:** **Baseline characteristics of case history and clinical examination in 75 dogs bitten by** ***Vipera berus***

**Parameter**	**PRED (n = 38)**	**PLACEBO (n = 37)**
Age (mean, years)	3.5	4.5
Weight (mean, kg)	21.6	21.2
Males/females	21/17	16/21
Bitten in the head region/limb	31/7	31/6
Median time (range) elapsed from bite (hours)	2.75 (1–24)	2.25 (0.5-11.5)

The dogs were treated with 1 mg/kg bodyweight prednisolone (PRED) or saline (PLACEBO) subcutaneously.

### Clinical assessments

The PRED group did not differ significantly (p > 0.05) from the PLACEBO group on Day 1 or Day 2 regarding mental status, extent of edema, decreased appetite, or presence of vomiting (Table 2). The appetite was not assessed on Day 1. Cardiac arrhythmia could be detected by auscultation in one dog in the PRED group on Day 1, and in one dog in the PLACEBO group on Day 2.

**Table 2:** **Clinical parameters in dogs bitten by** ***Vipera berus***

**Variable**	**Day**	**PRED n = 38**	**PLACEBO n = 37**
Reduced mental status	1	33/38 (87%)	27/37 (73%)
	2	10/38 (26%)	7/37 (19%)
Edema^1^	1	1.24 (n = 17)	1.22 (n = 18)
	2	1.21 (n = 17)	1.23 (n = 20)
Mild edema	1	2/34	8/33
Mild or no edema	2	12/34	16/34
Decreased appetite	2	10/34 (29%)	10/35 (29%)
Vomiting	1	6/38 (16%)	4/37 (11%)
	2	4/38 (11%)	2/37 (5.4%)

^1^Edema was compared to the circumference of the measured area at re-examination after 10–28 days. The number of dogs with available data on edema is given within brackets.

The dogs were treated with 1 mg/kg bodyweight prednisolone (PRED) or saline (PLACEBO) subcutaneously.

The proportion of dogs that developed diarrhea during the study period did not differ significantly (p = 0.2) between the PRED (5/33, 15%) and the PLACEBO (9/32, 28%) group. Duration of hospitalization did not differ between the two groups (median duration 2 days).

### Clinicopathological variables

#### CRP

**Figure 1:**
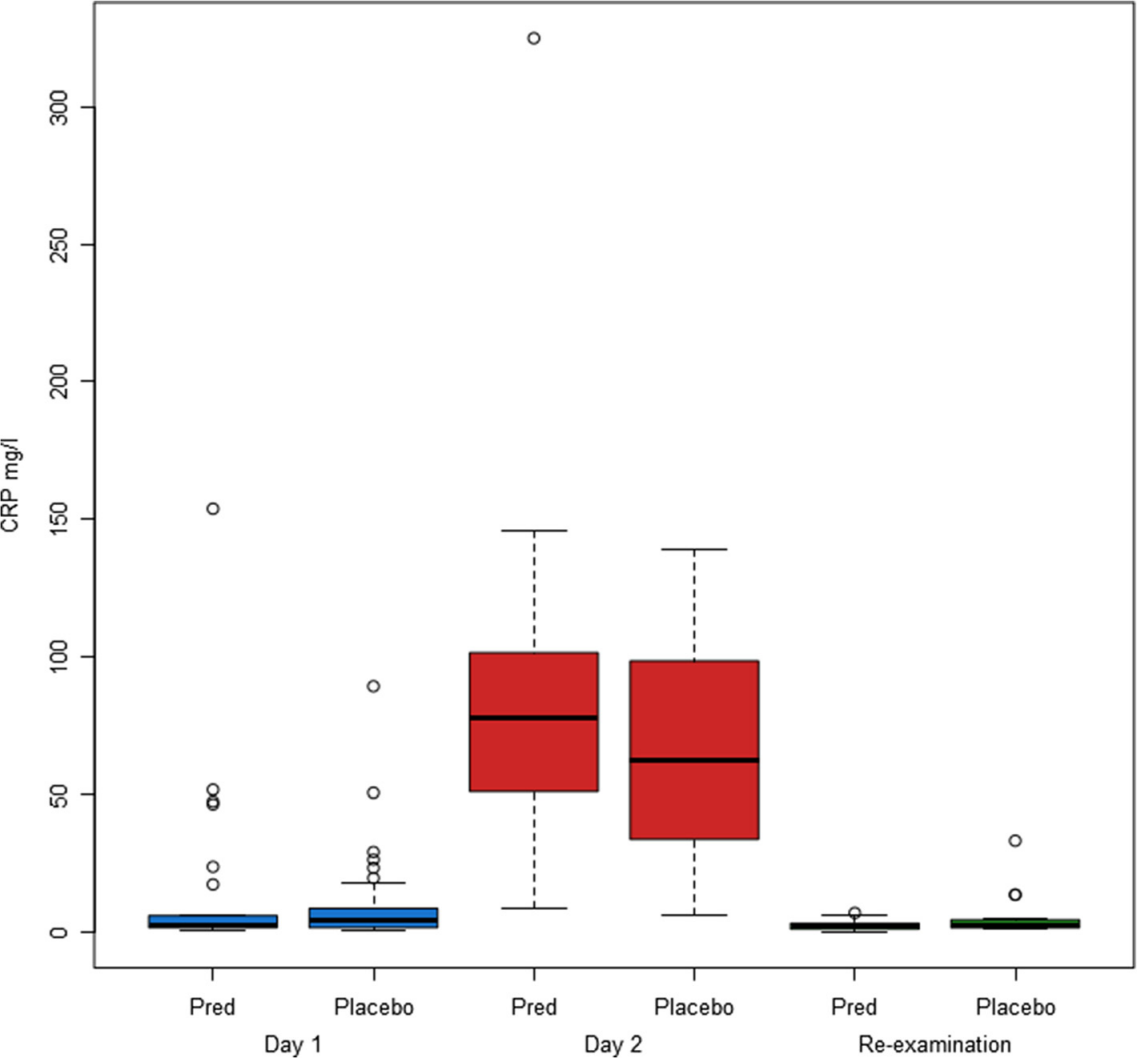
**Concentrations of C-reactive protein (CRP) in dogs bitten by V** ***ipera berus*** **.** Concentrations are shown as box plots. Dogs are treated with 1 mg/kg prednisolone (PRED) or saline (PLACEBO) subcutaneously.

CRP concentration did not differ significantly between the PRED and PLACEBO group on Day 1 (p = 0.59), Day 2 (p = 0.41) or at Re-exam (p = 0.26) (Figure 1).

#### Prothrombin time (PT) and hematology

The groups did not differ significantly (p > 0.05) in PT, hematocrit or platelet numbers on any of the sampling occasions. The two dogs with findings of abnormal PT concentration were both treated with prednisolone (PRED group).

The total leucocyte counts did not differ significantly between the groups on Day 1, Day 2 or at Re-exam (p > 0.05). Differential counts did not differ between groups, except for dogs in the PRED group with a significantly higher monocyte count on Day 2 (mean 0.9 × 10^9^/l) than dogs in the PLACEBO group (mean 0.4 × 10^9^/l, p < 0.01).

#### Cardiac troponin I (cTnI)

Concentrations of cTnI were significantly higher for dogs in the PRED group compared to dogs in the PLACEBO group on Day 1 (0.032 vs 0.015 μg/L, p = 0.04), but not on Day 2 or at Re-exam. The increase in cTnI concentration between Day 1 and Day 2 did not differ significantly between the groups (p = 0.9) (Table 3).

**Table 3:** **Median troponin concentration in dogs bitten by** ***Vipera berus***

	**PRED**	**PLACEBO**
Day 1 (μg/l)	0.03	0.015*
Day 2 (μg/l)	0.056	0.035
Re-Exam	0.015	0.012

*Statistically significant difference (p < 0.05) between groups (Mann–Whitney *U*-test).

Dogs were treated with 1 mg/kg prednisolone (PRED) or saline (PLACEBO) subcutaneously.

## Discussion

In the present study, clinical and clinicopathological parameters in dogs bitten by *V. berus* are described and the association of markers for systemic inflammation to clinical status investigated. In addition, to our knowledge, this is the first randomized double-blind and placebo-controlled clinical trial investigating the effect of prednisolone treatment in dogs bitten by *V. berus*.

No dog died during the study period, and the disease progression was benign with a mental status returning to normal or mildly reduced in all dogs during the first two days after the bite. Vomiting and diarrhea was described in 23/75 dogs (31%). Gastrointestinal signs have previously not been reported frequently in snake-bitten dogs, but are the most common signs of systemic *V. berus* envenomation in humans [9,10,12]. Cardiac arrhythmia was an uncommon finding in this study (2 dogs). One reason for cardiac arrhythmia being a rare finding could be that dogs treated with antivenom were excluded and at the animal hospitals participating, treatment with antivenom is often started when arrhythmia is detected. Cardiac arrhythmia after snake bite with *V. berus* is mainly of ventricular origin [8] and usually develops within 24 h of arrival [4].

Inflammation is part of the pathogenesis after snake envenomation [2,7]. In the current study, presence of inflammation was confirmed by the finding of increased CRP concentrations in most dogs (80%), as well as a left shift in neutrophils in about 25% of the dogs. Previous studies have shown that CRP is a quantitative marker of inflammation [7,19], which is in accordance with the findings in our study of a positive correlation between degree of edema swelling and CRP concentration. The CRP concentration did not differ between dogs with normal or decreased mental status, which may suggest that mental status mainly is affected by factors other than inflammation. An additional finding in this study was that the CRP concentration was often within the normal reference range at admission despite the presence of swelling and a markedly increased CRP concentration at the examination 24 h later (Day 2). However, after an inflammatory stimulus, an increase in blood CRP concentration occurs within 4–6 h, reaching the maximum concentration after approximately 24 h [7,19]. Therefore, a low CRP concentration in a snake bitten dog can be expected if the dog is sampled within only a few hours after the bite occurred.

Cardiac troponin is a biomarker for myocardial cell damage and cTnI has been studied previously in dogs bitten by *V. berus* [7,8]. In the present study, the cTnI concentration was higher in dogs with systemic inflammation than in dogs without systemic inflammation. Concentrations of cTnI and CRP were positively correlated on Day 2. These findings support the hypothesis by Langhorn et al. [7], that systemic inflammation plays a role in the pathogenesis of myocardial injury in dogs bitten by *V. berus*. The finding of hemoconcentration as found here on Day 1 has also been described in other studies of snake bite in dogs [2], and is likely to be caused by dehydration, third space loss of fluid or catecholamine-induced splenic contraction.

In the clinical trial, no effect of a single dose of prednisolone 1 mg/kg bw SC was detected on the main outcome variables selected, i.e. mental status and edema. This prednisolone dose was selected based on the Swedish recommendations for treatment of dogs bitten by *V. berus*, to get an antiinflammatory but not an immune-suppressive effect [20]. The lack of a clear positive effect of glucocorticoid treatment on clinical signs is in accordance with the findings in a prospective but nonrandomized previous study of 53 dogs bitten by *V. berus* given various but mostly single doses of glucocorticoid [4]. The effect of higher or repeated doses has hitherto not been studied. In humans, large retrospective studies have shown that treatment with corticosteroids is of no benefit after envenomation by *V. berus* [9,10,12] and also in children bitten by green pit vipers (*Cryptelytrops albolabris and Cryptelytrops macrops*), 1 mg/kg of prednisolone given orally for 3 days had no effect on edema reduction [21]. There are some concerns that treatment with glucocorticoids could decrease the effect of antivenom [22,23]. The effect of glucocorticoids on antivenom treatment in dogs bitten by *V. berus* has not been studied.

Since the introduction of antivenom, treatment with corticosteroids has decreased in humans, as has the incidence of extensive edema [10]. Treatment with antivenom is the only specific treatment for snake bite [24], and based on the results of the present study it seems reasonable to conclude that routine use of glucocorticoids in dogs is not necessary, although selected cases, e.g. with allergic reactions against the snake venom, may benefit from glucocorticoids. In addition, treatment with prednisolone did not affect any of the investigated clinicopathological parameters, except for dogs in the PRED group with a higher monocyte count on Day 2 than dogs in the PLACEBO group. This may be a coincidental finding because manual counting of monocytes is not precise [25] and the mean difference between the two groups was only 0.5 × 10^9^/l. It was considered less likely that the higher monocyte count in the PRED group was part of a stress leucogram caused by treatment because compared to the PLACEBO group there was no increased neutrophil count or decreased lymphocyte or eosinophil count, which has been reported earlier after treatment with high doses of glucocorticoids [26]. The lack of a stress leucogram could be explained by the observation that the leucogram is normal 24 h after a single dose of glucocorticoid [27].

This study has limitations. The presence of fang marks or observation of a snake was often a fact but not an absolute criterion for inclusion in the study, but dogs with untypical signs, where the veterinary surgeon in charge had concerns regarding the true presence of a snake bite, have not been included. The risk for the diagnosis of snake envenomation to be incorrect is therefore small. Most dogs were first-opinion patients and representative of dogs bitten by *V. berus* in Sweden, but for practical reasons, further treatment at the Animal Hospital was needed for inclusion in the study. Dogs with mild and only local signs were therefore often excluded. Eleven out of 140 dogs at Hospital 1 were not included because the owner declined to participate, and the degree of envenomation was not recorded. It can thus not be excluded that severely envenomated dogs were over-represented among these. Still, ten of the included dogs had a severe edema. We consider the dogs participating in the study as representative of dogs given supportive therapy in animal hospitals after *V. berus* envenomation.

Assessment of mental status, edema and other clinical parameters is subjective, especially when no objective criteria were set up beforehand. However our results, based on examinations by experienced veterinary surgeons, should be reasonably accurate. Some blood samples were missing, especially on Day 2 and from Animal Hospital 2, and 21 dogs were lost to follow-up. Yet, because both animal hospitals are referral centers in their area, we believe that the animal hospitals would have been contacted if the statuses of the dogs had worsened.

## Conclusion

In 75 dogs bitten by *V. berus* within the previous 24 hours, frequent initial findings were reduced mental status and decreased appetite. Development of gastrointestinal signs was common but all dogs recovered and no dog died during the study period. A single dose of prednisolone 1 mg/kg bw subcutaneously did not affect clinical or clinicopathological parameters. Snake bite induced systemic inflammation, defined as CRP > 35 mg/L, was detected in 80% of the dogs studied.

## Endnotes

^a^Prednisolonacetat-Injektionssuspension, CP Pharma, Burgdorf, Germany.

^b^Sarstedt AG & Co, Numbrecht, Germany.

^c^Medonic CA 620/530 Vet Analyzer, Boule Medical AB, Stockholm, Sweden.

^d^Sysmex XT-2000iV, Sysmex Corporation, Kobe, Japan.

^e^Gentian cCRP, Gentian AS, Moss, Norway.

^f^Abbott Architect c4000, Abbott Park, IL, US.

^g^ADVIA Centaur CP TnI-ultra, Siemens Healthcare, Diagnostics, Inc., Tarrytown, NY, US.

^h^Normotest, Nordic Diagnostica AB, Billdal, Sweden.

## Abbreviations

CRP: C-reactive protein
cTnI: Cardiac troponin I
IV: Intravenous
SC: subcutaneous
PLACEBO: Dogs treated with 1 mg/kg saline subcutaneously
PRED: Dogs treated with 1 mg/kg prednisolone subcutaneously
PT: Prothrombin time
Re-exam: Follow-up examination after 10–28 days
Bw: Body weight
*V. berus*: *Vipera berus*
